# Impact of Spousal Mental Illness on Healthcare Utilization Among Patients With Gastrointestinal Cancer

**DOI:** 10.1002/jso.27860

**Published:** 2024-09-05

**Authors:** Mujtaba Khalil, Selamawit Woldesenbet, Sidharth Iyer, Zayed Rashid, Abdullah Altaf, Erryk Katayama, Odysseas P. Chatzipanagiotou, Kristen M. Carpenter, Timothy M. Pawlik

**Affiliations:** ^1^ Department of Surgery The Ohio State University Wexner Medical Center and James Comprehensive Cancer Center Columbus Ohio USA; ^2^ Department of Psychiatry and Behavioral Health The Ohio State University Columbus Ohio USA

**Keywords:** cancer, caregiver burden, healthcare utilization, informal caregiver, mental illness, spouse

## Abstract

**Background:**

Informal caregiving involves increased responsibilities, with financial and emotional challenges, thereby affecting the well‐being of the caregiver. We aimed to investigate the effect of spousal mental illness on hospital visits and medical spending among patients with gastrointestinal (GI) cancer.

**Methods:**

Patients who underwent GI cancer surgery between 2013 and 2020 were identified from the IBM Marketscan database. Multivariable regression analysis was used to examine the association between spousal mental illness and healthcare utilization.

**Results:**

A total of 6,035 patients underwent GI surgery for a malignant indication. Median age was 54 years (IQR: 49–59), most patients were male (*n* = 3592, 59.5%), and had a CCI score of ≤ 2 (*n* = 5512, 91.3%). Of note, in the 1 year follow‐up period, 19.4% (anxiety: *n* = 509, 8.4%; depression: *n* = 301, 5.0%; both anxiety and depression: *n* = 273, 4.5%; severe mental illness: *n* = 86, 1.4%) of spouses developed a mental illness. On multivariable analysis, after controlling for competing factors, spousal mental illness remained independently associated with increased odds of emergency department visits (OR 1.20, 95% CI 1.05–1.38) and becoming a super healthcare utilizer (OR 1.37, 95% CI 1.04–1.79), as well as 12.1% (95% CI 10.6–15.3) higher medical spending.

**Conclusion:**

Among patients with GI cancer spousal mental illness is associated with higher rates of outpatient visits, emergency department visits, and expenditures during the 1‐year postoperative period. These findings underscore the importance of caregiving resources and counseling in alleviating caregiver burden, thereby reducing the overall burden on the healthcare system.

## 1 Introduction

A cancer diagnosis is a traumatic event that has a psychological impact on both the patient and their support system, including informal caregivers [1]. Informal caregivers include spouses, children, friends, or neighbors who provide uncompensated care, usually at home [2]. Of note, informal caregivers play a vital role in patient management by assisting with activities of daily living, providing emotional support, and coordinating care [3]. As such, informal caregiving requires emotional, physical, and financial investment which may have negative consequences on the caregiver's overall well‐being [4]. For instance, Greenwell et al. reported that caregiver burden is associated with motor symptoms, cognitive impairment, and disrupted sleep [5]. Moreover, cancer is increasingly being recognized as a chronic disease, with patients spending less time in the hospital and receiving most of their complex treatments in outpatient settings [6, 7]. In turn, informal caregivers are facing increasing duties and responsibilities, which may impact their own as well as the patient's physical and mental health [8].

The National Alliance for Caregiving reported that approximately 4 million people in the United States are providing care for an adult cancer patient, constituting approximately 8% of all caregivers nationwide [9]. In most cases, spouses are the primary informal caregivers for cancer patients [9, 10]. Of note, the risk of anxiety, stress‐related disorders, and depression increases by 30% in the first year following a cancer diagnosis [10]. Moreover, if the patient has advanced disease or a poor prognosis, the risk for spousal mental illness (MI) increases [10]. In turn, the mental health condition of the spouse may influence outcomes and the quality of life of patients [11, 12]. For instance, Rashid et al. studied individuals with Parkinson's disease and reported that spousal MI was associated with worse patient quality of life and more annual emergency department visits [13].

Patients with gastrointestinal cancer often undergo extensive surgery followed by radiotherapy and chemotherapy, which can leave these individuals frail [14]. Consequently, the responsibilities of the spousal caregiver may increase, potentially leading to MI and adverse patient outcomes [15]. Nevertheless, the effect of spousal MI on patient healthcare utilization and medical spending remains poorly defined. Therefore, the current study sought to define the impact of spousal MI on outpatient visits, emergency department visits, and medical spending among patients with gastrointestinal cancer.

## 2 Methods

### 2.1 Data Source, Study Population, and Cohort Selection

The IBM MarketScan Commercial Claims and Encounters database was queried using International Classification of Diseases (ICD) and Current Procedural Terminology (CPT) codes to identify patients who underwent gastrointestinal surgery for a malignant indication. The database encompasses 43–55 million employees across over 150 companies spanning all 50 states [16]. A notable strength of the IBM MarketScan database is the availability of an encrypted family identifier, which allows for the linking of all individuals covered by the same primary beneficiary, including their relationships to the primary insurance holder [16]. The analytic cohort included patients aged ≥ 18 who underwent esophagectomy, gastrectomy, hepatectomy, pancreatectomy, colectomy, and proctectomy for cancer between 2013 and 2020. Patients and their spouses were continuously enrolled in insurance for at least 12 months before the surgery and for 12 months postsurgery. Patients were excluded if they did not have a spouse in the database or had multiple spouses during the study period (e.g., due to divorce and remarriage). For patients who underwent multiple procedures during the study period only the first procedure was considered. The Institutional Review Board at Ohio State University approved this study and waived the requirement for informed consent since the data was deidentified.

### 2.2 Exposure

The primary exposure was MI in the spouse. ICD‐9‐CM and ICD‐10‐CM diagnosis codes were utilized to identify at least one outpatient visit for a MI by the spouse within the year following gastrointestinal cancer surgery in the index patient. MI was defined as depression, anxiety, bipolar disorder, schizophrenia, or other psychotic disorders [17]. Among these conditions, bipolar disorder, schizophrenia, and other psychotic disorders were further categorized as severe MI [17].

### 2.3 Covariates and Outcome of Interest

Baseline covariates included patient age, sex, insurance type, employment status (i.e., active full‐time, retiree active, and other), geographical region (i.e., Northeast, Northcentral, South, West), Charlson Comorbidity Index (CCI), and cancer type. Patient health insurance types included comprehensive health coverage, exclusive provider organization, health maintenance organization (HMO), point‐of‐service (POS), preferred provider organization (PPO), and other (consumer‐directed health plans and high‐deductible health plans). Spouse characteristics included age, sex, and MI.

The primary outcomes of interest included outpatient visits, emergency department visits, healthcare utilization, and cost of care in the year following cancer surgery. The cost was comprised of the total amount paid by the payer and patient for inpatient, outpatient, and pharmacy during the year following the cancer surgery. All costs were adjusted to 2020 US dollars using the medical care component of the consumer price index. A previously established method was utilized to identify super and low utilizers based on cost data [18]. Patients were categorized into four groups using a bisecting *k*‐means clustering method, with bin sorting by median to compute the cluster seed. This method assumed that there would be four distinct healthcare utilizer groups. Medical costs were first grouped into high and low observations, and then separated the high observations into their respective high and low groupings, followed by separating the low observations into their respective high and low groupings. The groups with the highest and lowest median annual expenditures were defined as super and low utilizers, respectively.

### 2.4 Statistical Analysis

The data were presented as median with interquartile range (IQR) for continuous variables and frequency (%) for categorical variables. Continuous variables were compared using the Wilcoxon rank‐sum test, while categorical variables were assessed using the Chi‐square or Fisher's exact tests, as appropriate. To visualize the expected expenditure of a healthcare episode over time, we constructed a 4‐week moving average. This analytic approach has been previously described and used in time series and healthcare cost analyses [18, 19]. Multivariable logistic regression analyses were used to examine the relationship between spousal MI and emergency department visits, as well as healthcare utilization; odds ratios (OR) and 95% confidence intervals (CIs) were reported. A multivariable generalized linear (GLM) regression model with a gamma distribution and a log link was fitted to examine total 1‐year expenditures. The models were adjusted for patient age, sex, CCI, employment status, region, insurance type, postoperative complications, extended length of hospital stay, cancer type, and year. Statistical analyses were performed using SPSS version 28 (IBM Corporation) and SAS version 9.4 (SAS Institute, Inc.). All tests were two‐sided, and a *p*‐value < 0.05 was considered statistically significant.

## 3 Results

### 3.1 Baseline Characteristics of Patient and Spouse

A total of 6,035 individuals underwent gastrointestinal surgery for a malignant indication (esophagus: *n* = 206, 3.4%; stomach: *n* = 302, 5.0%; liver: *n* = 126, 2.1%; bile duct: *n* = 120, 2.0%; pancreas: *n* = 460, 7.6%; colon: *n* = 3,549, 58.8%; rectum: *n* = 1,272, 21.1%). Median age was 54 years (IQR: 49–59), most patients were male (*n* = 3592, 59.5%) and had a CCI score of ≤ 2 (*n* = 5512, 91.3%). A majority of the patients resided in the South (*n* = 2701, 44.8%) or the North central (*n* = 1370, 22.7%), followed by the Northeast (*n* = 1136, 18.8%) and West (*n* = 812, 13.7%) regions of the United States. At the time of cancer diagnosis, 71.2% (*n* = 4301) of patients were employed full‐time, while 9.8% (*n* = 590) were retired. The most common health insurance type was PPO (*n* = 3294, 54.6%) followed by Other (*n* = 1386, 23.0%), HMO (*n* = 729, 12.1%), and POS (*n* = 317, 5.2%). In the 1‐year follow‐up period, median number of outpatient visits was 47 (IQR: 24−71), median expenditures were $143,160 (IQR: $84 337–$227 597), and 38.0% (*n* = 2292) of patients visited the emergency department (Table 1).

**Table 1:** Characteristics of patients and spouses by mental illness status.

Characteristics	Total (*n* = 6035)	Spouse without MI (*n* = 4866, 80.6%)	Spouse with MI (1169, 19.4%)	*p*‐value
**Patient**				
Age	54 (49–59)	54 (49−59)	54 (48−59)	0.054
Sex				<0.001
Male	3592 (59.5)	2722 (55.9)	870 (74.4)	
Female	2443 (40.5)	2144 (44.1)	299 (25.6)	
CCI				0.669
≤2	5512 (91.3)	4448 (91.4)	1064 (91.0)	
>2	523 (8.7)	418 (8.6)	105 (9.0)	
Insurance plan				0.179
Comprehensive	250 (4.1)	201 (4.1)	49 (4.2)	
EPO	59 (1.0)	52 (1.1)	7 (0.6)	
HMO	729 (12.1)	584 (12.0)	145 (12.4)	
POS	317 (5.2)	245 (5.0)	72 (6.2)	
PPO	3294 (54.6)	2680 (55.1)	614 (52.5)	
Others	1386 (23.0)	1104 (22.7)	282 (24.1)	
Employment status				0.012
Active full time	4301 (71.2)	3496 (71.8)	805 (68.8)	
Retiree	590 (9.8)	483 (10.0)	107 (9.2)	
Other	1144 (19.0)	887 (18.2)	257 (22.0)	
Geographical region				0.197
Northeast	1136 (18.8)	905 (18.6)	231 (19.8)	
North Central	1370 (22.7)	1092 (22.4)	278 (23.8)	
South	2701 (44.8)	2194 (45.3)	507 (43.3)	
West	812 (13.7)	659 (13.7)	153 (13.1)	
Cancer type				<0.001
Esophagus	206 (3.4)	147 (3.0)	59 (5.0)	
Stomach	302 (5.0)	228 (4.7)	74 (6.3)	
Liver	126 (2.1)	109 (2.2)	17 (1.5)	
Bile duct	120 (2.0)	100 (2.1)	20 (1.7)	
Pancreas	460 (7.6)	361 (7.4)	99 (8.5)	
Colon	3549 (58.8)	2896 (59.5)	653 (55.9)	
Rectum	1272 (21.1)	1025 (21.1)	247 (21.1)	
Spouse				
Age	56 (50−61)	56 (51–61)	55 (49–60)	<0.001
Sex				
Male	2443 (40.5)	2143 (44.0)	300 (25.7)	<0.001
Female	3592 (59.5)	2723 (56.0)	869 (74.3)	
Mental illness				—
Anxiety	509 (8.4)	—	509 (43.5)	
Depression	301 (5.0)	—	301 (25.7)	
Anxiety and depression	273 (4.5)	—	273 (23.4)	
Psychosis	86 (1.4)	—	86 (7.4)	

Abbreviations: EPO, exclusive provider organization; HMO, health maintenance organization: POS, point of service; PPO, preferred provider organization.

In the first year following a gastrointestinal cancer diagnosis, 1169 (19.4%) spouses visited the hospital for MI. Median age of the spouses was 56 years (IQR: 50–61), and the majority were female (*n* = 3592, 59.5%). The most common MI was anxiety (*n* = 509, 8.4%), followed by depression (*n* = 301, 5.0%), concomitant anxiety and depression (*n* = 273, 4.5%), and severe MI (*n* = 86, 1.4%). Of note, spouses who were younger (MI: 55 years [IQR: 49–60] vs. no MI: 56 years [IQR: 51–61]), and female (MI: 74.3% vs. no MI: 56.0%) were more likely to develop a MI (both *p* < 0.001). Moreover, spouses of patients with esophageal (MI: 5.0% vs. no MI: 3.0%), gastric (MI: 6.3% vs. no MI: 4.7%), or pancreatic cancer (MI: 8.5% vs. no MI: 7.4%) were more likely to develop a MI (all *p* < 0.001) (Table 1).

### 3.2 Effect of Spousal MI on Patients' Healthcare Utilization and Expenditures

**Figure 1:**
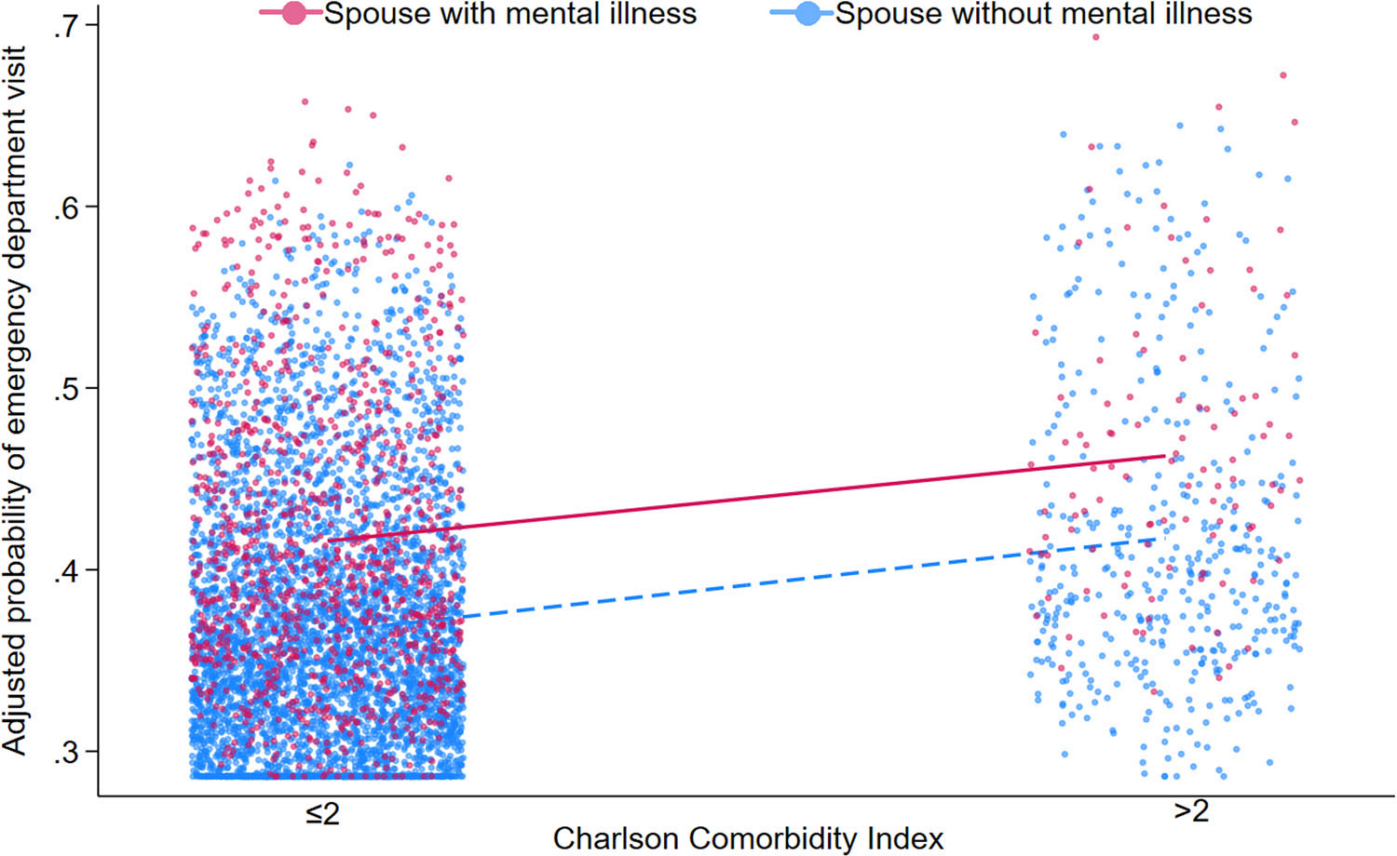
Scatterplot demonstrating the adjusted probability of emergency department visits, stratified by spousal mental illness.

Patients with spouses who had an MI were more likely to visit emergency departments (spouse with MI: 42.0% vs. spouse without MI: 37.0%) and outpatient clinics (spouse with MI: 53 visits [IQR: 29–74] vs. spouse without MI: 46 visits [IQR: 23–70]) (both *p* < 0.001). Consequently, 1‐year expenditures were higher among patients with a spouse who had MI (spouse with MI: $159 623 [IQR: $90 573–$253 699] vs. spouses without MI: $140 174 [IQR: $82 401–$220 725]; *p* < 0.001). Moreover, patients with a spouse who had MI were more likely to become superutilizers (spouse with MI: 9.5% vs. spouse without MI: 7.0%) (Table 2). On multivariable analysis, after controlling for competing factors, spousal MI remained independently associated with increased odds of emergency department visits (OR 1.20, 95% CI 1.05–1.38), and becoming a super utilizer of healthcare (OR 1.37, 95% CI 1.04–1.79), as well as with 12.1% (95% CI 10.6–15.3) higher medical spending (Figure 1 and Table 3).

**Table 2:** Health and financial outcomes of patients by spousal mental illness status.

Outcomes	Total (*n* = 6,035)	Spouse without MI (*n* = 4866, 80.6%)	Spouse with MI (*n* = 1,169, 19.4%)	*p*‐value
Discharge disposition				0.396
Home/self‐care	4594 (76.1)	3722 (76.5)	872 (74.6)	
Home health services	1040 (17.2)	822 (16.9)	218 (18.6)	
Other	401 (6.7)	322 (6.6)	79 (6.8)	
Emergency department visit	2292 (38.0)	1801 (37.0)	491 (42.0)	0.002
Outpatient visit	47.0 (24.0–71.0)	46.0 (23.0–70.0)	53.0 (29.0–74.0)	<0.001
1‐year total expenditures, $	143 160 (84 337–227 597)	140 174 (82 401–220 725)	159 623 (90 573–253 699)	<0.001
Healthcare utilization				0.014
Low	2350 (38.9)	1916 (39.4)	434 (37.1)	
Average	1777 (29.4)	1449 (29.8)	328 (28.1)	
High	1455 (24.1)	1159 (23.8)	296 (25.3)	
Super	453 (7.6)	342 (7.0)	111 (9.5)	

**Table 3:** Multivariable logistic regression examining the association between spousal mental illness and patient's health outcomes.

Outcomes	OR (ref: spouse without MI)	95% CI	*p*‐value
Emergency department visit	1.20	1.05–1.38	< 0.006
Super utilization	1.37	1.04–1.79	< 0.001

**Figure 2:**
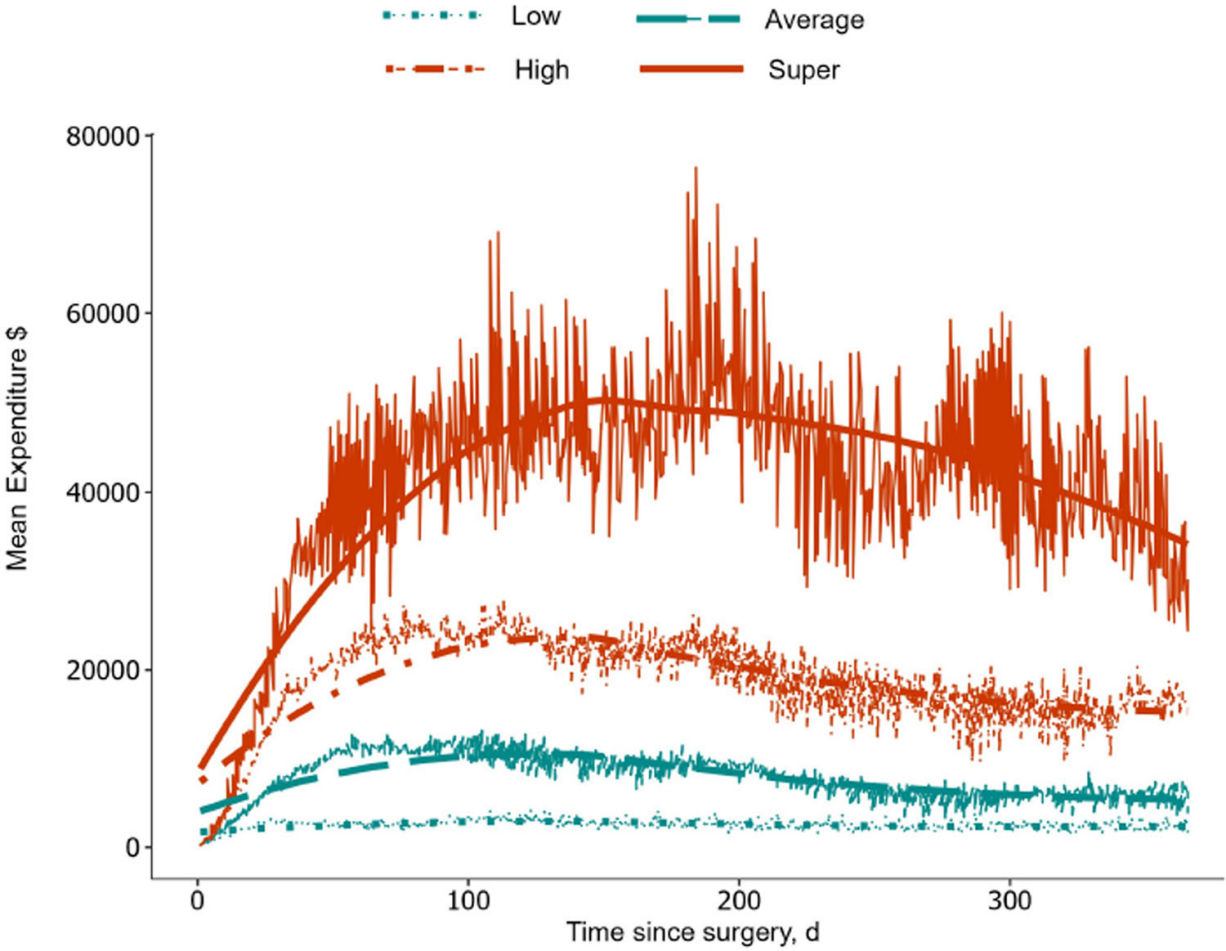
Moving averages for low, average, high, and super‐utilizers.

**Figure 3:**
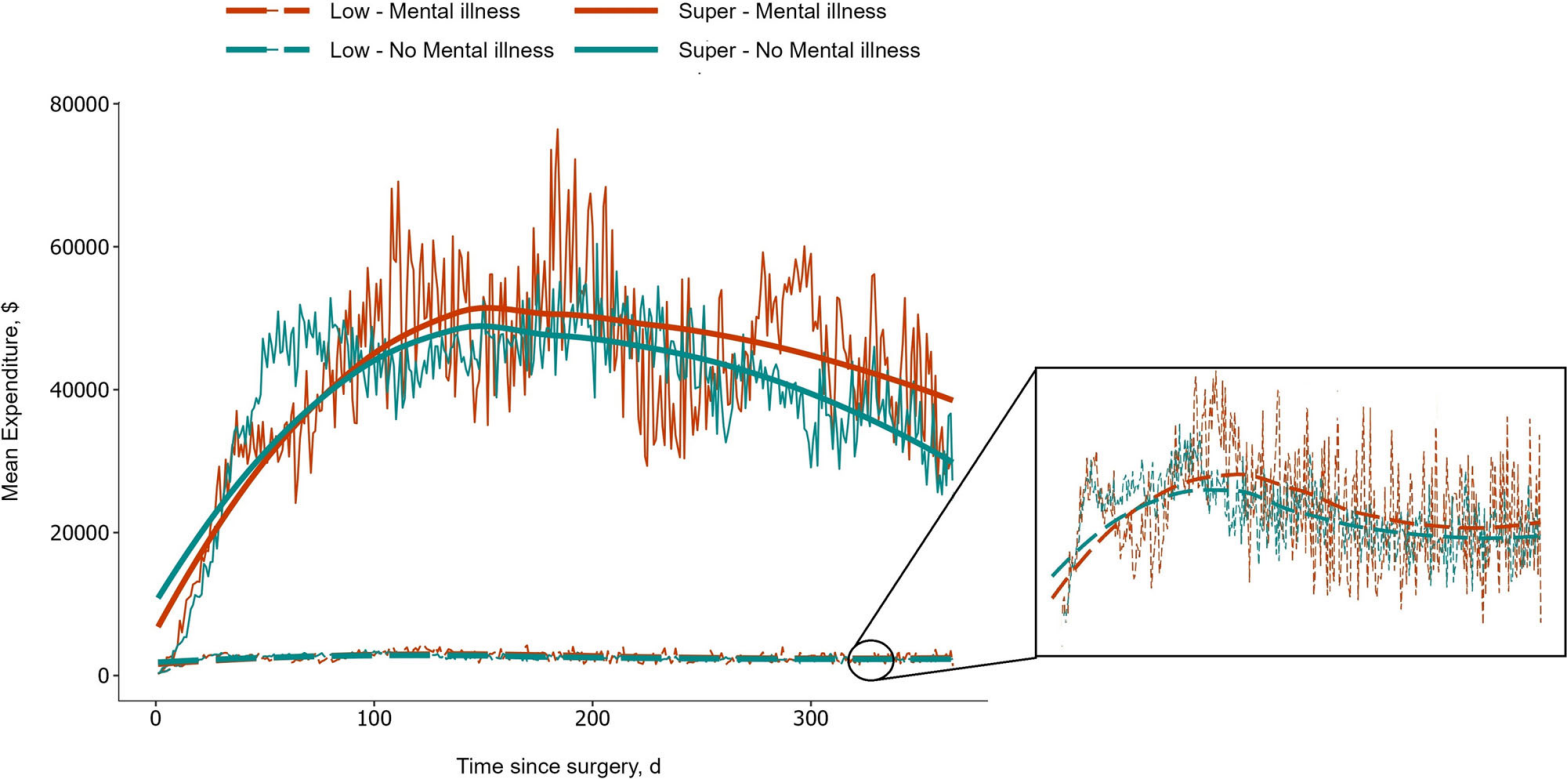
Moving averages for low and super‐utilizers, stratified by spousal mental illness.

Patients were stratified into four clusters based on healthcare utilization (low: *n* = 2350, 38.9%; average: *n* = 1777, 29.4%; high: *n* = 1455, 24.1%; super: *n* = 453, 7.6%) (Figure 2 and Table 2). The moving expenditure average demonstrated that following gastrointestinal cancer surgery, medical spending of super‐utilizers increased exponentially, reaching a peak before experiencing a gradual decline. In contrast, for low‐utilizers, medical spending remained relatively stable throughout the year. When stratifying patients based on the MI status of their spouses, individuals with a spouse who had MI exhibited higher medical spending. This trend was consistent among both high and low utilizers (Figure 3).

## 4 Discussion

Informal caregivers are an invaluable resource who support financial and emotional needs and provide day‐to‐day care for individuals with cancer [3]. Of note, informal caregiver presence is associated with fewer complications, better symptom control, delayed nursing home placement, and better quality of life [20]. However, cancer is a chronic disease and requires long‐term financial and emotional investment from the patient and adjacent support system [6, 7]. As such, informal caregiving can lead to adverse mental health consequences [15]. Notably, the risk of anxiety, stress‐related disorders, and depression increases by 30% in spouses during the first year following a cancer diagnosis [10]. Nonetheless, the effect of spousal MI on patient hospital visits and healthcare utilization remains poorly defined. Therefore, the current study was important in addressing this important gap by utilizing a national database to evaluate the association of spousal MI on outpatient visits, emergency department visits, and healthcare utilization among patients with gastrointestinal cancer. Of note, spousal MI was associated with increased outpatient and emergency department visits among patients with gastrointestinal cancer during their 1‐year follow‐up period. Moreover, patients with a spouse experiencing MI had 37% higher odds of becoming a super healthcare utilizer. The current data underscore the importance of caregiving resources and counseling in reducing spousal MI, thereby improving the outcomes of patients with gastrointestinal cancer.

Patients with gastrointestinal cancer represent a vulnerable population who experience frequent hospital visits [21]. Previous studies have estimated that between 30% and 50% of individuals with cancer visit the emergency department at least once annually, a rate consistent with the findings of the current study [21, 22]. Additionally, we noted a spousal MI incidence of 19.4%, which aligned with prior literature [10]. Although the association of spousal MI on outcomes and quality of life has been studied in chronic medical conditions such as dementia and Parkinson's disease, to our knowledge this is the first study to investigate the association with emergency department visits and healthcare utilization among patients with gastrointestinal cancer [13, 23]. Importantly, the current study noted that spousal MI was associated with an increase in both annual emergency department and outpatient visits. These findings may be attributed to the uncertainty surrounding the disease and various social and household factors [4, 5]. Patients with gastrointestinal cancer often are treated with surgery followed by chemotherapy and radiotherapy, which may leave them frail [14]. In turn, the responsibilities of informal caregivers can increase as patients require assistance with medication and activities of daily living [4]. Moreover, caring for patients with cancer demands a significant amount of time, along with financial and emotional investment [5]. Consequently, when caregivers lack social support or emotional reserves, it may result in burnout, impacting their mental health [5]. Therefore, the caregiver may struggle to meet the progressively challenging needs of the patient, leading to more frequent visits to the outpatient or emergency department [13].

This rise in hospital visits carries several financial implications for both caregivers and the healthcare system [24, 25]. Of note, the current study utilized a novel statistical method to identify super‐utilizers in the 1‐year follow‐up and noted that spousal MI was associated with 37% higher odds of becoming a super‐utilizer. Among patients with cancer, over half of emergency department visits are due to preventable causes such as pain, fever, difficulty breathing, nausea, vomiting, and dehydration [22, 26]. Importantly, these visits can be avoided with improved symptom management and family education [22, 26]. Furthermore, once admitted to the emergency department, these patients often undergo an extensive workup due to the complexity of their underlying disease, incurring substantial costs [26]. In sum, spousal MI may represent a risk factor for patients with gastrointestinal cancer to become a healthcare superutilizer. It is crucial, therefore, that targeted efforts are made to improve spousal mental health [15]. These efforts may potentially improve the caregiver's ability to care for the patient and ultimately reduce the financial burden on both the family and the healthcare system [23].

It is vital to understand and mitigate mental health disorders among spouses of individuals with cancer [15]. Although studies have reported that MI among caregivers may be associated with the level of care burden, poor social support systems, and low education, initiatives aimed at supporting caregivers' mental health are still in the early stages [20, 27]. Importantly, physicians should provide a safe space for open communication to allow spouses to express their feelings and fears without judgment [28]. Moreover, offering additional information about diagnosis and treatment options can alleviate some anxiety surrounding the uncertainty of cancer [29]. Providing resources and support services for the spouse, such as counseling, support groups, or respite care, may help them manage their burnout and stress [30]. For instance, the American Cancer Society offers online communities and local support groups, where spouses of cancer patients can connect with others facing similar challenges and find emotional support [30]. Spouses should also be encouraged to practice self‐care through activities such as guided relaxation, regular exercise, and establishing healthy routines [27]. Furthermore, offering practical assistance with daily tasks may alleviate their burden, while also reminding spouses of the importance of self‐care amidst their caregiving responsibilities [28]. When needed, spouses should be referred to professional counseling services to help process emotions and find better coping strategies [10].

Despite several strengths, including a large, nationally representative patient cohort, the findings of the current study should be interpreted considering a few limitations. The IBM MarketScan database includes individuals with employer‐sponsored health insurance, and the results may not be generalizable to individuals who are uninsured or are insured through a government program. Moreover, employer‐sponsored insurance coverage is only extended to married partners, thus the results may not be applicable to those in nonmarital partnerships. Mental health conditions are generally underreported in databases [31]. Therefore, the number of spouses identified with a mental health illness may not be a true representation of the general population.

In conclusion, among patients with gastrointestinal cancer spousal MI was associated with increased outpatient and emergency department visits during the 1‐year follow‐up period. Moreover, gastrointestinal cancer patients with a spouse experiencing MI had higher odds of becoming super‐utilizers. These findings underscore the importance of spousal mental health care and counseling to alleviate caregiver burden, which may represent a means to reduce the overall healthcare burden of patients with cancer.

## Conflicts of Interest

The authors declare no conflicts of interest.

## Disclosure

The authors have nothing to report.

## Synopsis

Spousal mental illness is associated with higher rates of outpatient visits, emergency department visits, and expenditures during the 1‐year postoperative period. These findings underscore the importance of caregiving resources and counseling in alleviating caregiver burden, thereby reducing the overall burden on the healthcare system.

## Data Availability

The data for this study were obtained from the IBM MarketScan database. There are restrictions to the availability of this data, which is used under license for this study. Data can be accessed with permission from the IBM MarketScan Commercial Database.
